# Rethinking the Multidimensionality of Growth Mindset Amid the COVID-19 Pandemic: A Systematic Review and Framework Proposal

**DOI:** 10.3389/fpsyg.2022.572220

**Published:** 2022-07-01

**Authors:** Yun-Ruei Ku, Catanya Stager

**Affiliations:** Educational Studies in Psychology, Research Methodology, and Counseling University of Alabama, Tuscaloosa, AL, United States

**Keywords:** growth mindset, resilience, higher education, emotion regulation, mental health, self-regulation, self-efficacy

## Abstract

Students, staff, and faculty in higher education are facing unprecedented challenges due to the COVID-19 pandemic. Recent data revealed that a good number of academic activities and opportunities were disrupted as a result of the COVID-19 pandemic and its variants. While much uncertainty remains for the next academic year, how higher education institutions and their students might improve responses to the rapidly changing situation matters. This systematic review and framework proposal aim to update previous empirical work and examine the current evidence for the effectiveness of growth mindset interventions in young adults. To this end, a systematic search identified 20 empirical studies involving 5, 805 young adults. These studies examined growth mindset within ecologically valid educational contexts and various content areas. Generally, these findings showed that brief messages of growth mindset can improve underrepresented students' academic performance and facilitate other relevant psychological constructs. In addition, we argue, although growth mindset has been identified as a unitary concept, it is comprised of multiple interdependent skills, such as self-control, self-efficacy, and self-esteem. Understanding the nature of growth mindset may contribute to successful mindset implementation. Therefore, this article presents a practical framework to help educators in higher education rethink the multidimensionality of growth mindset and to provide their students with alternative routes to achieve their goals. Finally, additional articles were discussed to help evaluate growth mindset interventions in higher education.

## Introduction

Students' growth mindsets—how malleable they perceive their abilities to be—play a pivotal role in their academic achievement (Dweck, 2006; Yeager and Dweck, 2020) and have been linked to multiple positive outcomes, including improved mental health (Schroder et al., 2017b), decreased stress (Burnette et al., 2020a) and even brain activity (Schroder et al., 2014). The extent to which and how growth mindset modulates one's achievement has been descriptively examined but rarely systematically explored (but see Cheng et al., 2021 for a review). The current systematic review and framework proposal aims to identify previous empirical work and examine the current evidence for the effects of growth mindset interventions in young adults, and to propose a practical framework that introduces educators of today to better support their learners.

Currently, academics around the world are facing the ongoing impact of an unprecedented situation—COVID-19, which has been disrupting academic activities and in-person interactions (García-Morales et al., 2021; Rodríguez-Planas, 2022). These unexpected challenges may be chronic stressors and long-term risk factors to retention and graduation goals, especially for at-risk college students (Rodríguez-Planas, 2022). In such difficult, yet unavoidable moments, mindset can play a crucial role in the future trajectory of learning (Schroder et al., 2017a). Behavioral strategies and cognitive trainings (or interventions) for enhancing growth mindset have gained increased attention (Broda et al., 2018), in part due to rising levels of anxiety in young adults (Schroder et al., 2019).

The first aim of this paper is to update previous empirical work and examine the current evidence for the effects of growth mindset interventions in young adults of higher education. In addition, this paper examines unique themes that the studies address and commonalities shared. The second aim of this study aims to contend that growth mindset is not a unitary concept but rather is multifaceted. The overarching concept of growth mindset may be too broad and fail to capture the nuances of the sub-abilities of growth mindset. Additionally, the concept of growth mindset may also be too somewhat abstract to implement in practice. Therefore, developing and maintaining the sub-abilities associated with growth mindset can be seen as prerequisites to successful learning and psychological wellbeing. Our proposal adds to the current literature in that we have not only suggested instructional strategies but also distinguished between the relevant sub-abilities in terms of how they differ and how each concept can be instilled in young adults.

By understanding the underpinnings of growth mindset sub-abilities, researchers and educators can better focus on ways to develop more successful interventions. Furthermore, educational institutions can then be positioned to provide students with the support they need to flourish and succeed despite periods of physical isolation and limited access to traditional psychotherapies (Hood et al., 2021).

### What Is Growth Mindset?

A growth mindset embodies the thought that one's personal attributes, such as intelligence, skill, and ability can be developed and improved through effort. Mindset theory (Dweck, 1946; Dweck and Leggett, 1988) was originally derived from attribution theory and achievement goal theory in motivational research. Specifically, mindset effects are meaningfully heterogeneous across persons and situations (see Yeager and Dweck, 2020 for a discussion). Mindsets are typically evaluated by gauging individuals' agreement or disagreement with some statements on his or her capability to change or thrive in the face of challenges. Importantly, individuals can be at different points of the continuum at different times.

Compared to cognitive ability, this non-cognitive skill asserts that intelligence can be malleable (i.e., incremental theory) as opposed to fixed (i.e., entity theory). This malleability of mindset has been demonstrated to encourage people to think flexibly so they are not discouraged as easily in challenging situations (Dweck, 2006). A major question in social sciences is why some people tend to persist after failure and frustration while others succumb to setbacks immediately and never recover. Motivation has been closely associated with implicit theories of intelligence (Dweck, 2006). Implicit theories of intelligence entail a belief that personal characteristics such as one's intelligence and ability can be changed and developed later in life through effort and hard work. Such mental state is frequently called “growth mindset.” However, individuals with a fixed mindset do not think that human traits and characteristics can be intervened with and altered. In other words, personal traits and characteristics are pre-determined and innate (see Dweck, 2006 for details). Relevant studies investigated underlying beliefs these students have about intelligence and learning. These individuals' views of themselves have been shown to ultimately influence their academic performance, motivation, behavior, and self-efficacy (Rhew et al., 2018).

Incremental implicit theories are closely associated with a growth mindset (Dweck et al., 1995). It is proposed that people can either embrace an entity theory or an incremental theory of their personal characteristics. Individuals with an entity theory think that they are incapable of changing most of their personal traits (e.g., strengths). On the contrary, individuals with an incremental theory would expect changes and improvements in their personal traits (Dweck and Leggett, 1988). Moreover, individuals with an entity theory or a fixed mindset seem to emphasize performance goals or outcomes of their actions. By contrast, those with an incremental theory or a growth mindset tend to focus on learning goals or processes, which has been considered to increase learner motivation (Dweck, 1986). Thus, learners with a growth mindset tend to be more intrinsically motivated to learn and to apply different strategies to learning compared to learners with a fixed mindset (Yan et al., 2014). However, it should be noted that growth mindset and fixed mindset are just the two ends of a continuum, which means that individuals can possess varying degrees of a mindset under different circumstances (Haimovitz and Dweck, 2017). It is important to note that mindset can be dynamic, ever-changing, and subjective although it has been assessed in a more static manner. The following review addresses growth mindset interventions and explores its effects on the outcome measures.

### Why Is It Important?

Since the outbreak of the COVID-19, educational interventions have attracted increased attention in higher education (Adedoyin and Soykan, 2020; Yeo et al., 2021). Growth mindset interventions are often considered light-touch interventions (Kim et al., 2022) and are a common intervention to address both education (2022) and mental health needs related to the pandemic (Mosanya, 2021). In the current study, we examined the effectiveness of the growth mindset intervention and its contexts of implementation within higher education.

A growth mindset intervention explicitly teaches that individual's academic and intellectual abilities have the potential to develop and grow. A growth mindset intervention encompass the instructional delivery and/or discussion of potential concrete actions and strategies a learner can take to implement a growth mindset (Yeager and Dweck, 2020).

Growth mindset research develops out of the social cognitive theory (Bandura, 1986) and growth mindset has also been related to implicit theories of intelligence and even terms such as self-efficacy. The conceptual framework, reciprocal interactions, was proposed by Bandura (1986) to explain the dynamic interactions between environmental inputs (e.g., instruction), personal processes (e.g., social comparisons), and behavioral influences (e.g., effort). For example, the iterative process between perceived progress, self-efficacy, and goal pursuit is critical for student motivation and learning (Schunk and DiBenedetto, 2020). Similarly, since online and asynchronous instruction do not function in the same fashion as do in-person contexts, students' autonomy to plan for learning requires increased level of self-regulation. In other words, they need to set proper goals, select strategies, and metacognitively monitor their cognitive processes during learning (e.g., Miele and Scholer, 2017). Growth mindset messages have been shown to foster self-regulation in various contexts, such as substance use (Burnette et al., 2013, 2019a).

A long-term tele-education has adversely impacted traditional higher education and is likely to pose long-term impacts beyond the pandemic. Various instructional platforms ultimately led to a new teaching model that combined online teaching and autonomous learning (Canning et al., 2019; Sharma and Bhaskar, 2020). A recent study showed that the growth mindset could contribute to college students' learning engagement through the roles of perceived COVID-19 event strength (i.e., one's perception of external events) and perceived stress (i.e., one's feeling of internal pressure) during the COVID-19 pandemic. These factors have been suggested to promote sustained learning and positive emotion toward learning (Zhao et al., 2021).

The objective of this study is to answer the following three research questions:

1. What is currently known about the benefits and limitations of the growth mindset intervention in higher education?
2. What is the strength of the evidence in support of these findings?
3. What are the implications of these studies for instructional practices?

In addition to reviewing substantive findings regarding the growth mindset intervention, this review also aims to advance the understanding of relevant theories and practices and propose a practical framework for intervention implementation.

## Methods

This project adheres to the QUOROM statement (Moher et al., 1999) and the Preferred Reporting Items for Systematic Reviews and Meta-Analyses (PRISMA) guidelines for conducting systematic reviews (Moher et al., 2009, 2015).

### Literature Search

We completed a search in four major electronic databases (APA PsycInfo, Web of Science, Scopus, and Pubmed) for articles up through January 2022 (i.e., no date restrictions) related to a combination of search terms relating to growth mindset, implicit theories of intelligence, population age (college and university students), intervention (e.g., educational intervention), and the type of study. All articles were filtered for article type (peer-reviewed), language of study (English), and publication (non-book chapters). Database specific filters were also applied if available. Table 1 shows our specific search terms with article results by database.

**Table 1:** Search terms used in article search across four databases.

**Database**	**Field**	**Boolean search terms**	**Article results**
APA PsycInfo	Abstract	(University or college or higher education) and (growth mindset or implicit theories of intelligence or self regul*) and intervention	277
NCBI PubMed	Abstract/Title *filtered by age 19–24	[(growth AND mindset) OR implicit theor* OR (self AND regulat*) AND [University OR college OR (higher AND education)] AND intervention [Title/Abstract]]	496
Scopus	Abstract	ABS (university OR college OR higher AND education) AND (growth AND mindset OR implicit AND theories AND of AND intelligence OR self-reg?) AND intervention	221
Web of science	Abstract	AB = [(university OR college OR higher education) AND (growth mindset OR implicit theories of intelligence OR self-reg*) AND intervention]	526

### Inclusion and Exclusion Criteria

These database searches initially led to 1,520 relevant abstracts, and after removing 280 duplicates, 1,240 abstracts remained. For the remaining 1,240 abstracts, the lead researchers applied the eligibility criteria to identify relevant studies. Inclusion criteria included studies that (a) used empirical designs; (b) appeared in peer-reviewed journals; (c) were available in the English language; (d) involved growth mindset as a primary or secondary variable; (e) set within a higher education/college/university context and (f) included an intervention for growth mindset. The intervention or training should have been explicitly developed in line with the incremental theory of intelligence (Dweck and Leggett, 1988). Excluded articles included unpublished studies, gray literature such as business reports, conference proceedings, editorial comments, announcements, review articles (including previous systematic reviews), and single-case studies. Additionally, other documents such as dissertations, case studies, book chapters, conference proceedings, reviews, or meta-analyses whose main independent variable was grit (Duckworth et al., 2007) or other closely relevant factors, were excluded from the article list if there was no mindset outcome.

#### Study Selection

**Figure 1:**
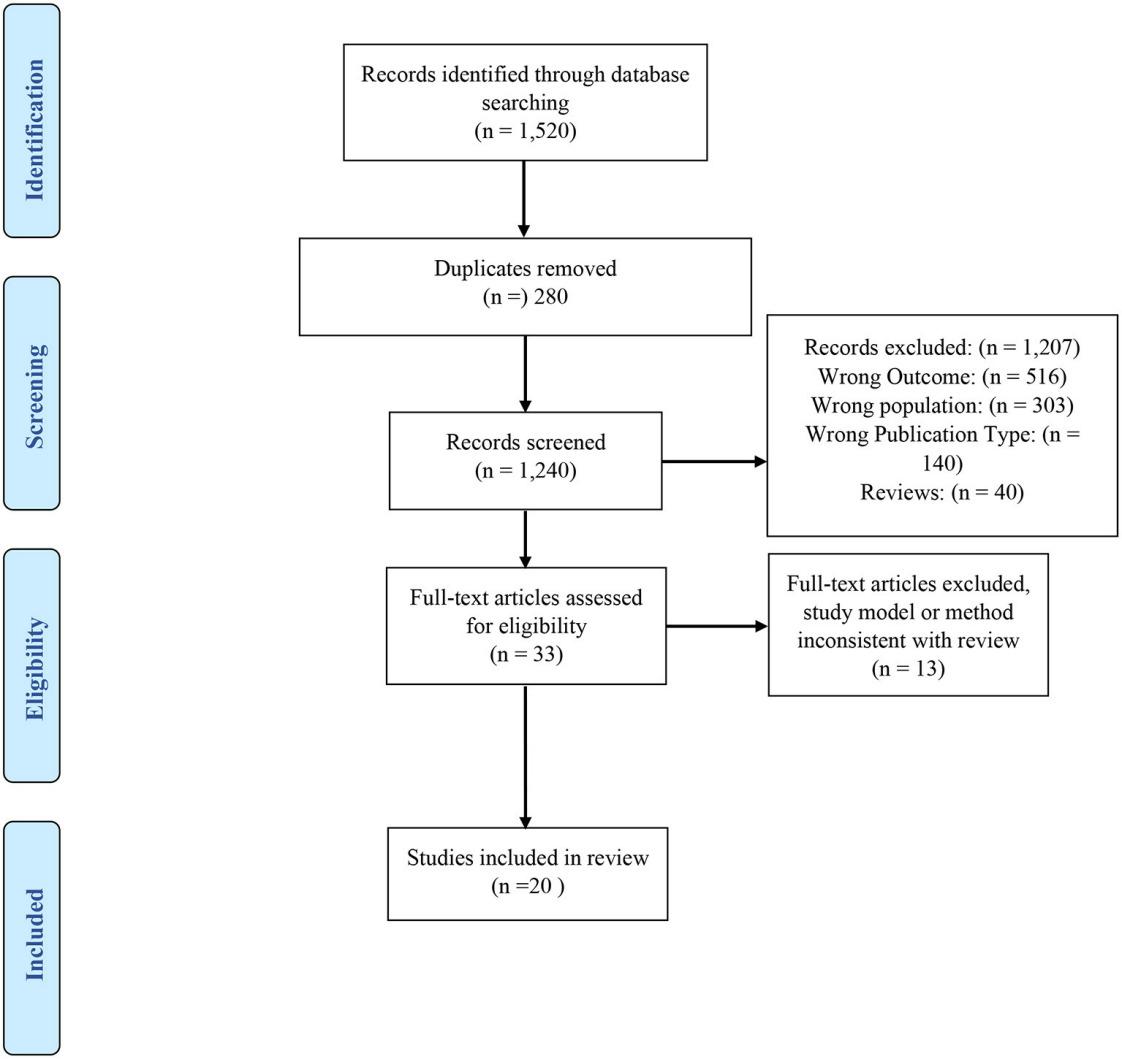
PRISMA flow diagram of results and publication selection.

The included titles and abstracts from our initial search were reviewed by both authors. Each reviewer blindly and independently reviewed each article's title and abstract and made a decision to exclude or include based upon the eligibility criteria. Once all reviews were complete, the authors un-blinded the independent reviews and resolved all conflicts through consensus. This reduced the list to 20 articles. See Figure 1 for the PRISMA flow diagram (Moher et al., 2009).

#### Reporting Criteria

Once the final 20 articles were selected based on the inclusion and exclusion criteria, the two authors independently and methodically extracted data from each study and reconciled the findings by consensus. Specifically, we extracted data related to (a) sample, (b) delivery, (c) intervention, (d) duration, and (e) outcome measures (Table 2).

**Table 2:** Overview of the selected studies.

**References**	**Sample**	**Delivery**	**Intervention**	**Duration**	**Outcome measures**
Brez et al. (2020)	Undergraduate students enrolled in gateway math and psychology courses across three academic terms (Mindset *n* = 1,314)	Randomly assigned (online) to either control or experimental group	Participants were randomly assigned to a fixed or growth mindset group to read an article about how the brain can/cannot grow stronger through effort and how difficult subjects such as math can/cannot be mastered and that anyone can learn math (or other subjects)	One-time	Course grade, term GPA, and term credit hours earned
Broda et al. (2018)	First-year incoming undergraduate students (Mindset *n* = 2,135)	Randomly assigned (online) to either control or experimental group	Participants in the mindset intervention group read a short scientific article on brain plasticity, indicating that intelligence is not fixed and can be improved with effort. After reading the article, students are asked several reflective questions in which they identify moments in life when they may have adopted a growth mindset	One-time	Grade point average (GPA), course credits attempted, course credits completed, full-time enrollment, cumulative GPA
Burnette et al. (2019b)	Undergraduate students enrolled in introductory computer science courses (Mindset *n* = 245)	Randomly assigned to either control or experimental group	Participants in the mindset intervention group read a short scientific article about brain plasticity, or the idea that the brain, similar to other muscles, can grow. Afterwards, participants were asked several reflective questions in which they are encouraged to identify moments in their own lives when they may have or have not adopted a growth mindset	Multiple sessions across a semester	High school GPA, ACT/SAT scores, Pell grant eligibility, and first-generation status
Cheng et al. (2021)	Graduate students (Mindset *n* = 11)	Randomly assigned to either control or experimental group	A set of infographics was developed with different topics for each week (i.e., narratives and visual images)	Weekly, over 6 weeks	Implicit theories of intelligence scale, written reflections
Daniels et al. (2020)	Undergraduate pre-service teachers (Mindset *n* = 99)	Randomly assigned to beliefs-only, approaches-only, combined beliefs and approaches, or control condition	Participants read a scientific article that contained accurate content about mindsets and emphasized teachers' capabilities to enhance student motivation and growth mindset	Multiple sessions across different terms	A set of post-intervention questionnaires (e.g., teachers' personal responsibility for student motivation)
Derr and Morrow (2020)	Undergraduate students (Mindset *n* = 30)	Randomly assigned to either one of the learning tasks that were designed to promote a growth mindset of personality (personality condition) or a matching exercise designed to promote a growth mindset of athleticism (athleticism condition) or control condition	Participants read an article designed to enhance individuals' endorsement of growth-oriented statements about personality	One-time	Defender self-efficacy, moral disengagement, and perceived defender behavior
Fink et al. (2018)	Undergraduate students enrolled in general chemistry (Mindset *n* = 275)	Pseudo-randomly to either control or experimental group	Participants read a related short article online and completed a comprehension quiz, wrote a refection how the intervention influences their perception and behavior	3 1-h lectures per week during the first semester	Final-exam score, written responses to the phase 2 and 3 reflection prompts
Frey et al. (2018)	First-year undergraduate students enrolled in general chemistry (Mindset *n* = 278)	Randomly assigned to either control or experimental group	Students self-administered three doses of the intervention as part of their online homework	3 1-h lectures per week during the first semester	Final-exam score
Glerum et al. (2018)	Students enrolled in a vocational program (Mindset *n* = 22)	Randomly assigned to either control or experimental group	Watched three related short video clips and two post-videos short writing exercises	Multiple sessions over 10 weeks	Math test performance, implicit theories of intelligence scale
Goldstein et al. (2021)	Predominantly first-year undergraduate students in aerospace engineering, civil & environmental, industrial & systems engineering, and mechanical science and engineering (*n* = 296)	Within-subject design	The workshop modules were concurrent with the courses and mirroring what was covered in each course. The workshop contains seven diverse topics designed to improve students' spatial visualization skills. For each topic, students were required to complete a set of online exercises.	A total of 3.5-h of a workshop that trained students in visual-spatial thinking over 7 weeks	Growth mindset for spatial intelligence, Purdue Spatial Visualization Tests
Hacisalihoglu et al. (2020)	First-year undergraduate students enrolled in the college of science and technology's Scientist Life Skills course (*n* = 79)	Within-subject design	The course included videos and professional workshops on the concepts of growth mindset, grit, and critical thinking	Participants attended 2 weekly classes that included case studies, essays, and a final project (150 min in total)	Online mindset questionnaire, grit questionnaire, critical thinking assessment, and academic ranking
Lewis et al. (2020)	Community college registered nursing students (*n* = 35)	Within-subject design	A presentation on neuroplasticity, a brief video of an expert, discussion with peers, learning strategies that students could adopt to cultivate a growth mindset	An one-hour educational session	Williams Inventory of Learning Strategies (WILS) tool, including mindset assessment
McCabe et al. (2020)	First-year students enrolled in one of the required courses (Mindset *n* = 119)	Randomly assigned to either control or experimental group	Online academic talks related to growth mindset and infographic materials over a course	Multiple sessions over a semester	Mindset assessment, educational enjoyment, academic importance assessment, GPAs, retention
McPartlan et al. (2020)	Predominantly first-year undergraduate students enrolled in biological sciences (Mindset *n* = 274)	Randomly assigned to control, social belonging, combined, or growth mindset group	Read materials related growth mindset, Written reflections	One-time	Implicit theories of intelligence scale, written reflections, grades, single-item belonging uncertainty assessment
Miller and Srougi (2021)	Third and fourth year undergraduate students enrolled in a one semester biochemistry course (Mindset n = 87)	Cohorts were assigned to either control or experimental group	A set of metacognitive course activities, including self-reflection exercises, concept mapping, growth minded messaging, exam wrappers, and instructor talk that tends to foster a growth mindset	Over a semester	Biochemistry diagnostic assessment, online homework and quizzes, written reflections, final exam grade
Mills and Mills (2018)	Undergraduate students enrolled in remedial math classes (Mindset *n* = 98)	Randomly assigned to either control or experimental group	Lessons taught the difference between growth and fixed mindsets. Lessons aimed to cultivate growth mindset, share stories of successful people. Participants were then asked to share stories of people that they knew who had persevered through adversity	4 weekly sessions (about 30 min each) over	Course grade, retention during the following semester, mindset assessment
Parada and Verlhiac (2021)	First-year undergraduate students enrolled in mandatory psychology course (Mindset *n* = 125)	Randomly assigned to control, original growth mindset, or revised growth mindset group	Participants read a scientific article describing intelligence and emphasizing its malleability and potential to grow through effort.	One-time	A set of questionnaires (e.g., achievement goal questionnaire, perceived stress, coping strategy), two-item mindset assessment
Samuel and Warner (2019)	First-year community college students enrolled in a two-semester developmental statistics course (Mindset *n* = 17)	Randomly assigned to either control or experimental group	Participants received a combination of mindfulness and growth mindset intervention (e.g., deep breathing session) and recited growth mindset messages	Multiple sessions over two semesters	A set of questionnaires and surveys (e.g., math anxiety and self-efficacy surveys), a subgroup of participants joined a focus group interview
Saraff et al. (2020)	First Year college students (Mindset *n* = 150)	Quasi-experimental non-randomization	Participants in one of the treatment groups (i.e., treatment 2 & 3) received mindfulness-based approach in developing the positive self-concept, self-esteem and growth mindset. In addition to the control group, treatment group 1 only discussed strategies of mindfulness. Treatment group 2 received meditation exercises along with mindfulness strategies	12 sessions of discussions and exercises based on meditation about 1-h	A set of scales (e.g., Self-concept Short Scale), implicit theories of intelligence scale
Torsney et al. (2021)	College and university students (*n* = 116)	Randomly assigned (online) to either control or experimental group	Participants were instructed to complete a packet of surveys at pretest and then engage in a series of activities, including reading a refutation text about the malleability of a student's intelligence	One-time (about 30 min)	Mindset scales, anticipated GPA for the semester, and open-ended questions asking their perception of intelligence

#### Assessment of Rigor

We also used the previously validated Checklist to Enhance Methodological Quality in Intervention Programs (Chacón-Moscoso et al., 2016) to better understand the methodological rigor of each study. This tool examines 12 characteristics of study quality, including inclusion and exclusion criteria for the units, experimental design, attrition, attrition between groups, exclusions after randomization, follow-up period, occasions of measurement, measures in pre-test appear in post-test, standardized dependent variables, control techniques, construct definition of outcome, and statistical methods for imputing missing data (see Table 3). In cases where the criteria were not discussed or assessed, we left the study “N/A” in the table. The two authors independently reviewed each included article using the assessment tool. Upon completion, they compared their results, and all conflicts were resolved by consensus.

**Table 3:** Methodological quality 12-item checklist.

**References**	**Inclusion and exclusion criteria** **for the units** **provided**	**Methodology or design**	**Attrition**	**Attrition** **between** **groups**	**Exclusions after randomization**	**Follow-up** **period**	**Occasions of measurement for each variable**	**Measures in pre-test appear in post-test**	**Standardized dependent variables**	**Control techniques**	**Construct definition of outcome**	**Statistical methods for imputing missing** **data**
Brez et al. (2020)	Yes	Experimental-Randomized	Yes	No	No	Yes	Pre- and post-intervention	All of them	Standardized questionnaires or self-reports	Masking (beneficiaries)	Replicable by reader in own setting	No
Broda et al. (2018)	Yes	Experimental-Randomized	Yes	Yes	Yes	Yes	Post-intervention only	N/A	Standardized questionnaires or self-reports	Masking (beneficiaries)	Replicable by reader in own setting	Yes
Burnette et al. (2020b)	Yes	Experimental-Randomized	Yes	Yes	Yes	Yes	Pre- and post-intervention	All of them	Standardized questionnaires or self-reports	Double-masking	Replicable by reader in own setting	Yes
Cheng et al. (2021)	Yes	Experimental-Randomized	Yes	Yes	Yes	Yes	Pre- and post-intervention	All of them	Standardized questionnaires or self-reports	Masking (beneficiaries)	Replicable by reader in own setting	N/A
Daniels et al. (2020)	Yes	Experimental-Randomized	Yes	Yes	Yes	Yes	Pre- and post-intervention	All of them	Standardized questionnaires or self-reports	Masking (beneficiaries)	Vague definition	N/A
Derr and Morrow (2020)	Yes	Experimental-Randomized	Yes	Yes	Yes	N/A	Pre- and post-intervention	All of them	Standardized questionnaires or self-reports	Masking (beneficiaries)	Replicable by reader in own setting	Yes
Fink et al. (2018)	Yes	Experimental-Randomized	Yes	Yes	Yes	Yes	Post-intervention only	N/A	Standardized questionnaires or self-reports	Masking (beneficiaries)	Replicable by reader in own setting	No
Frey et al. (2018)	Yes	Experimental-Randomized	Yes	Yes	Yes	Yes	Post-intervention only	N/A	Standardized questionnaires or self-reports	Masking (beneficiaries)	Vague definition	No
Glerum et al. (2018)	Yes	Experimental-Randomized	Yes	Yes	N/A	Yes	Pre- and post-intervention	All of them	Without (self-reports and *post-hoc* records)	Masking (beneficiaries)	Vague definition	N/A
Goldstein et al. (2021)	Yes	Pre-Experimental/Others	Yes	N/A	N/A	Yes	Pre- and post-intervention	All of them	Without (self-reports and *post-hoc* records)	Other (need to specify)	Replicable by reader in own setting	N/A
Hacisalihoglu et al. (2020)	Yes	Quasi-Experimental	N/A	N/A	N/A	Yes	Pre- and post-intervention	All of them	Without (self-reports and *post-hoc* records)	Other (need to specify)	Replicable by reader in own setting	N/A
Lewis et al. (2020)	Yes	Quasi-Experimental	N/A	N/A	N/A	Yes	Pre- and post-intervention	All of them	Without (self-reports and *post-hoc* records)	Other (need to specify)	Replicable by reader in own setting	N/A
McCabe et al. (2020)	Yes	Experimental-Randomized	Yes	Yes	Yes	Yes	Post-intervention only	All of them	Without (self-reports and *post-hoc* records)	Masking (beneficiaries)	Replicable by reader in own setting	N/A
McPartlan et al. (2020)	Yes	Experimental-Randomized	No	No	No	Yes	Pre- and post-intervention	All of them	Without (self-reports and *post-hoc* records)	Masking (beneficiaries)	Replicable by reader in own setting	No
Miller and Srougi (2021)	Yes	Quasi-Experimental	No	No	No	Yes	Pre- and post-intervention	All of them	Without (self-reports and *post-hoc* records)	Masking (beneficiaries)	Replicable by reader in own setting	N/A
Mills and Mills (2018)	Yes	Experimental-Randomized	Yes	Yes	Yes	Yes	Pre- and post-intervention	All of them	Standardized questionnaires or self-reports	Masking (beneficiaries)	Replicable by reader in own setting	N/A
Parada and Verlhiac (2021)	Yes	Experimental-Randomized	Yes	Yes	Yes	Yes	Pre- and post-intervention	All of them	Without (self-reports and *post-hoc* records)	Double-masking	Replicable by reader in own setting	N/A
Samuel and Warner (2019)	Yes	Experimental-Randomized	Yes	Yes	N/A	Yes	Pre- and post-intervention	Some	Without (self-reports and *post-hoc* records)	Masking (beneficiaries)	Replicable by reader in own setting	N/A
Saraff et al. (2020)	Yes	Quasi-Experimental	No	No	No	Yes	Pre- and post-intervention	All of them	Without (self-reports and *post-hoc* records)	Masking (beneficiaries)	Vague definition	N/A
Torsney et al. (2021)	Yes	Experimental-Randomized	Yes	Yes	N/A	Yes	Pre- and post-intervention	All of them	Without (self-reports and *post-hoc* records)	Masking (beneficiaries)	Replicable by reader in own setting	N/A

## Results

The search yielded 1,240 publications, of which 20 articles finally met the inclusion criteria. All included studies were published within the last 5 years (2018–2021).

### Study Participants

Eight studies examined the effects of growth mindset interventions on first-year undergraduate students enrolled in introductory courses (Frey et al., 2018; Samuel and Warner, 2019; Hacisalihoglu et al., 2020; McCabe et al., 2020; McPartlan et al., 2020; Saraff et al., 2020; Goldstein et al., 2021; Parada and Verlhiac, 2021). One study used graduate student sample (Cheng et al., 2021). One study used undergraduate students enrolled in remedial math course (Mills and Mills, 2018). One study used registered nurses (Lewis et al., 2020). One study used undergraduate students enrolled in a vocational program (Glerum et al., 2018). A portion of studies reported specific sample characteristics (e.g., different racial minorities, various socioeconomic statuses, and first-generation students). Finally, one study used undergraduate pre-service teachers (Daniels et al., 2020). A total of 5, 805 participants received a growth mindset intervention or an intervention that intends to cultivate a growth mindset (i.e., Saraff et al., 2020).

### Content Areas

Eleven out of the twenty studies assessed the effectiveness of growth mindset interventions within STEM (e.g., math, statistics, psychology, computer science, engineering, biology, biochemistry, and chemistry). Other studies did not specify its contexts in terms of content area. There is increasing interest today in how a brief mindset intervention may contribute to outcomes other than academic achievement (e.g., GPA). It is evident that researchers in different disciplines have started to recognize the value and the need of psychological interventions in addition to regular courses in higher education. Specifically, an increasing literature has examined the effectiveness of Wise interventions (WIs), which focuses on a single construct, often drawn from diverse psychological perspectives, and target specific psychological processes (O'Brien and Lomas, 2016; Walton and Wilson, 2018). WIs are generally brief and more focused, and thus can be easily incorporated into original curricula.

### Research Methods and Data Analyses

As can be seen in Table 1, different studies used very different research designs and outcome measures. Thirteen studies used only quantitative methods, whereas seven studies used mixed methods, such as written reflection (e.g., Fink et al., 2018; Cheng et al., 2021). This variability makes it difficult to meta-analyze these results. For example, two of the selected studies examined factors that might indirectly influence individuals' levels of growth or fixed mindsets using a path analysis. We might, therefore, observe important discrepancies in the effect sizes reported while we adopt a more rigorous means to summarize these results (Moreau, 2020). It is also important to consider qualitative results pertaining to the use and effectiveness of a growth mindset intervention in higher education (e.g., Limeri et al., 2020), such as self-reported psychological wellbeing, social belonging, and perception of peers.

### Materials and Delivery Methods

In general, most included studies used randomized controlled trial (RCT) design. Participants were randomly assigned to a growth mindset intervention or a matched control group. As mentioned before, one study used quasi-experimental non-randomization (Saraff et al., 2020) and three studies used within-subject design (i.e., no randomization). Most studies followed the guidelines reported in Yeager et al. (2016). Several studies modified the common intervention to meet their needs. For example, Derr and Morrow (2020) asked their participants to read a vignette of a peer student victimized by his or her peers, and then completed paper-and-pencil measures of defender self-efficacy, moral disengagement, and perceived defender behavior. The intervention has two versions that were identical with one exception; the control condition included an exercise designed to promote a growth mindset of athleticism while the experimental condition included an exercise designed to promote a trait growth mindset.

Another revision was reported in Torsney et al. (2021). Refutation texts were used as part of the growth mindset intervention. Refutation intends to introduce a misconception to students and help them identify scientifically inaccurate concepts and acquire evidence-based knowledge. Moreover, their findings suggested that refutation text along with other educational activities can change, at least momentarily, college students' implicit theories of intelligence. Ninety five percent of the included studies delivered the intervention materials through an online platform (e.g., learning management system).

## A Practical Framework For Practicing Growth Mindset

Post-secondary students are increasingly transitioning to an internet-based method of teaching and learning. While the general public becomes anxious, panic, or even depressed about the social isolation, students in higher education experience more uncertainty and hopelessness about their future. The new reality has forced students to change plans and adjust to the current situation. Every individual might attend to or interpret these stressors differently making emotion regulation strategies an urgent need (see Tabibnia and Radecki, 2018 for a review). Learning in higher education is not solely determined by individuals' personal characteristics, but also to the extent that the teachers able to support or hinder learning and motivation (Jeno, 2015). In the time of self-isolation, educational institutions and their instructors may bear an important responsibility for helping young learners adjust to both physical and psychological changes.

There may be college- or department-wide services for helping students access additional resources to reduce the impact of the pandemic on financial needs. However, supports for students' mental health seem sparse (Lee, 2020). Tabibnia and Radecki (2018) provides very useful and empirically tested behavioral strategies that enhance psychological resilience in the face of negative emotions. For example, recognizing one's own emotions and expressing them explicitly to their close friends and family members could significantly reduce the detrimental effects of negative emotions and thoughts. Universities could frequently track the wellbeing needs of their students over the different stages of the COVID-19 pandemic and encourage their learners to seek ways to make the best out of the situation.

The second part of this study includes a proposal for a practical framework for instructors in higher education to revisit the idea of growth mindset based on the findings reported here and to manage different sub-abilities that have been shown to be highly associated with growth mindset. In fact, growth mindset has become more of a general term suggesting that human abilities can be developed through effort (Dweck, 2006; Yeager and Dweck, 2020). Developing a more nuanced view of growth mindset in light of the sub-abilities undergirding it is one way that growth mindset can become easier to implement in educational settings.

**Figure 2:**
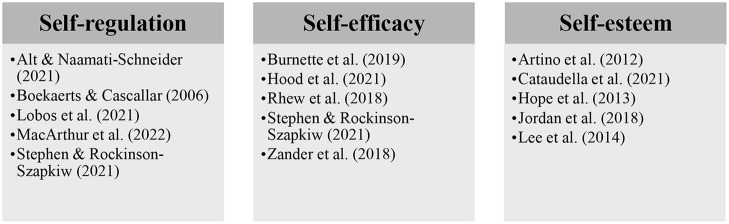
A practical framework for practicing growth mindset-related components & related references.

To educators, implementing growth mindset ideas in the curriculum can be dreadful and requires enormous energy and dedication. However, we propose that implementing growth mindset is too large of a goal. Instead, growth mindset can and should be considered as a multifaceted concept. By doing it, implementing growth mindset pedagogy becomes more manageable. In what follows, we identify three components of self-regulation, self-efficacy, and self-esteem, ones that have been frequently shown to be associated with growth mindset and are vital to its implementation in higher education (see Figure 2). Interested readers are encouraged to refer to these articles.

Boekaerts and Cascallar (2006) addressed some of the most critical questions concerning self-regulation, such as “What is self-regulated learning?,” “What strategies do students need to direct their own learning?,” “What environmental cues would trigger self-regulation strategies?,” and “What can teachers do to help student to self-regulate their learning?.” They provided very valuable and useful recommendations for instructional practices to promote self-regulation in young adults.

A significant number of previous findings have shown that self-esteem and/or self-efficacy are closely associated with growth mindset (e.g., Burnette et al., 2019b) or, at least, highly correlated with grade (e.g., Li and Bates, 2020). Self-efficacy is one's belief in their capacity to act to achieve desired outcomes (Bandura, 1997; Hope et al., 2013). Self-efficacy and self-esteem represent two different psychological variables and need to be managed differently. Generally, self-efficacy is more associated with motivational constructs than is self-esteem, whereas self-esteem is more associated with affective constructs (Chen et al., 2004; Jordan et al., 2018). Recent findings suggest that self-efficacy-focused curriculum is urgently needed when transitioning from traditional classrooms to other teaching and learning modalities (Talosa et al., 2021). By distilling growth mindset into these three core sub-abilities of self-regulation, self-efficacy, and self-esteem, educators can better focus their curricula and efforts on their desired outcomes (Zander et al., 2018).

Recent electrophysiological findings have shown that individuals possessing higher level of growth mindset attend to errors more often than those in a control group and have higher post-error accuracy (e.g., Schroder et al., 2014; also see Ng, 2018 for a review). Similarly, growth mindset has been shown to predict self-regulatory practices and strategies in the face of failures and challenges (see Burnette et al., 2013 for a review). Therefore, it is important to include self-regulation in the current proposal. Self-regulation can be conceptualized as goal-related processes, including goal setting, goal operating, and goal monitoring (Carver and Scheier, 2008). Self-regulatory strategies have been shown to be crucial to the initial development of a skill and individual performance in natural settings (Zimmerman, 1998). Educators in higher education can design their curricula accordingly to provide timely support and guidance to the students.

## Discussion

This study presented a systematic review of growth mindset interventions among higher education students and a proposed framework to explore the sub-abilities of growth mindset. Using multiple databases and specified key word searches, the authors narrowed the entirety of growth mindset literature studies to 20 studies that met the inclusion criteria. Those articles were then examined to answer three research questions.

Our first research question considered the benefits and limitation of growth mindset intervention literature in higher education populations. Our synthesis showed that growth mindset intervention is a brief and easily administered intervention for diverse content areas in higher education. The growth mindset intervention was easily integrated into instructional materials. However, we have identified several limitations of these selected papers. First, many studies do not include follow-up periods to determine how well-changes brought about by the growth mindset intervention endure over time or transfer to other contexts outside of the intervention. Most of the studies use a pre-post design, but few studies examined student mindset at a follow-up time point, either in school or afterwards. This is a serious limitation in that the purpose of the intervention is to empower students and take control over their own learning, which extends beyond the classroom, especially considering changes due to the pandemic. Thus, it would be desirable to know whether mindset changes remain after conclusion of the intervention. More research is needed to examine whether these interventional effects can be maintained and generalized to contexts outside of the instructional setting.

Our second research question examined the strength of the evidence of these findings. Our analysis discovered that many studies were lacking in statistical rigor. For example, almost 90% of the selected studies did not mention anything about the missing data. In other words, the authors did not report the presence of missing data during the data collection process and if they did, how did they address those missing data. Specifically, the validity of research inferences can be severely compromised due to improperly handling missing data (Lang and Little, 2016). In Table 3, most studies were labeled n/a for the column of missing data imputation. Since we did not even know whether these studies had missing responses in their data, we decided to label them under the not applicable category. We recommend that future studies clearly state the details about the missing data.

Our final research question considered the implications of these findings for instructional practice and implementation in higher education and reported thematic connections among the studies. Our findings suggest that growth mindset interventions have been used in science, technology, engineering, and math, with analyses ranging from quantitative to mixed methods to qualitative. Higher education interventions also include a wide variety of participants, including students representing education of undergraduates, graduates, and medicine. COVID-19 significantly disrupted in-person and face-to-face interactions. Consequently, virtual interventions have become increasingly common, and it is unclear how more or less effective virtual interventions are, in comparison to face-to-face interventions. It is still uncertain the extent of the effectiveness of virtual mindset interventions in the academic trajectory of students, after the intervention is complete. Not only has the way of instruction delivery changed, but also how people connect and communicate with one another. In this time of uncertainty, growth mindset, or broadly speaking, psychological resilience, is a critical priority.

In addition, growth mindset is not a unitary concept, and is associated with multiple sub-abilities, such as self-regulation and self-awareness (Mrazek et al., 2018). How might these abilities help us remain socially connected? Research has begun to address students' attitudes toward face-to-face vs. online instruction (e.g., Cole, 2016). In face-to-face learning, students could communicate with their instructors effectively through body language and gestures, whereas it seems more difficult with online instruction. Thus, students often experience frustration in the process of transitioning to online learning and continue to experience frustration afterwards (Cataudella et al., 2021). As a result, reflecting upon oneself (e.g., self-reappraisal strategy to reframe an event and respond differently) and exerting aforementioned abilities are essential in protecting faculty, staff, and students from chronic mental illnesses.

With signs that the spread of the pandemic is coming under control, it is time to prepare higher education systems for the new normal after the pandemic and its variants. Major changes and precautions might be in place immediately after campuses reopening. Therefore, educational institutions may need to provide more than one alternative for their students to learn, so that they would consider it as an opportunity to choose the most suitable and comfortable way to learn for them.

One of the benefits for having a growth mindset is that growth minded individuals tend to view mistakes as opportunities to learn and improve. Cognitive neuroscience has shown that growth minded individuals display higher post-error accuracy and reduced neural activity of emotional distress to mistakes made during the task (Moser et al., 2011). In the face of psychological and physical challenges, both educators and students need to re-examine the concept of growth mindset and cultivate their sub-abilities. If being growth minded is the ultimate goal, then developing these sub-abilities, or skills, is our immediate goal, as learning is a marathon, not a sprint.

As mentioned earlier, adaptive coping strategies are important and learnable. As psychological and neuroscientific research on growth mindset continues to accelerate, higher educational institutions play a key role in helping their students combat distress and recover from the current crisis. The new normal implies that campus life will never be the same for all of us. Under this circumstance, factors such as cultural sensitivity, socioeconomic status, gender equality, and financial downturn need to be more deeply and carefully considered.

Importantly, mental and physical illness can become chronic, resulting from high stress levels. Engaging in active coping strategies in response to the crisis may help to manage current and future stressors, which in turn encourages healthy mental and physical functioning. Simply put, in difficult times like this, educational institutions should bear more responsibility than ever to provide additional assistance to help their students avoid unhealthy choices (e.g., substance abuse). In this regard, growth mindset, or resilience in general, becomes more indispensable than ever. Institutional services and support should be implemented to help those students who have lost their usual routines and are stuck in chronic passivity to overcome barriers and succeed.

## Conclusion

As universities, colleges, and students plan and prepare for an unknown future, how might these educational institutions help students along the way to a successful career? A more positive and malleable way of interpreting our surroundings matters more than ever. While some students might have better self-regulatory skills, others do not. Therefore, higher education institutions can help their students identify additional resources, try new strategies, and seek input from others to promote resilience and their psychological needs over various stages of the pandemic. Likewise, students may use their time while learning remotely to develop the sub-abilities associated with growth mindset, so that they can maintain productivity and contribute to their institutions. Taken together, this paper hopes to summarize recent empirical findings about growth mindset interventions and conclude with practical considerations for application of the proposed framework to implement growth-mindset-related practices in higher education.

## Data Availability Statement

The original contributions presented in the study are included in the article/supplementary material, further inquiries can be directed to the corresponding author/s.

## Author Contributions

Y-RK conceived and designed the analysis. CS performed the analysis. Y-RK and CS wrote the manuscript. Both authors contributed to the article and approved the submitted version.

## Conflict of Interest

The author declares that the research was conducted in the absence of any commercial or financial relationships that could be construed as a potential conflictof interest.

## Publisher's Note

All claims expressed in this article are solely those of the authors and do not necessarily represent those of their affiliated organizations, or those of the publisher, the editors and the reviewers. Any product that may be evaluated in this article, or claim that may be made by its manufacturer, is not guaranteed or endorsed by the publisher.
